# Type I Interferons in COVID-19 Pathogenesis

**DOI:** 10.3390/biology10090829

**Published:** 2021-08-26

**Authors:** Enrico Palermo, Daniele Di Carlo, Marco Sgarbanti, John Hiscott

**Affiliations:** 1 Istituto Pasteur Italia—Cenci Bolognetti Foundation, Viale Regina Elena 291, 00161 Rome, Italy; daniele.dicarlo@istitutopasteur.it; 2 Department of Infectious Diseases, Istituto Superiore di Sanità, 00161 Rome, Italy; marco.sgarbanti@iss.it

**Keywords:** type I IFNs, innate immunity, SARS-CoV-2, COVID-19

## Abstract

**Simple Summary:**

The innate antiviral immune response is essential to limit virus replication at early stages of infection, thus preventing viral spread and pathogenesis. Nevertheless, viruses have evolved different strategies to evade innate immune control. In this review, we describe recent findings delineating the relationship between SARS-CoV-2 and type I IFN response in vitro and in vivo and report current studies using IFN-based therapy for COVID-19 treatment.

**Abstract:**

Among the many activities attributed to the type I interferon (IFN) multigene family, their roles as mediators of the antiviral immune response have emerged as important components of the host response to Severe Acute Respiratory Syndrome Coronavirus 2 (SARS-CoV-2) infection. Viruses likewise have evolved multiple immune evasion strategies to circumvent the host immune response and promote virus propagation and dissemination. Therefore, a thorough characterization of host–virus interactions is essential to understand SARS-CoV-2 pathogenesis. Here, we summarize the virus-mediated evasion of the IFN responses and the viral functions involved, the genetic basis of IFN production in SARS-CoV-2 infection and the progress of clinical trials designed to utilize type I IFN as a potential therapeutic tool.

## 1. Introduction

In December 2019, an outbreak of acute respiratory syndrome of unknown etiology was reported in Wuhan, China [1]. Soon thereafter, the severe acute respiratory syndrome coronavirus 2 (SARS-CoV-2) was identified as the causative agent of coronavirus infectious disease 2019 (COVID-19) and, in March 2020, the World Health Organization declared the COVID-19 outbreak a global pandemic [1,2]. As of 13 July 2021, the pandemic has accounted for over 210 million confirmed cases of COVID-19 worldwide, including more than 4 million deaths [3], together with an enormous social and economic impact throughout the world [4]. SARS-CoV-2 infection manifests with a broad spectrum of clinical patterns, resulting in asymptomatic cases in most individuals and inducing mild to severe illness in others, with fever, cough, headache and myalgia identified as common symptoms in moderate COVID-19, whereas severe pneumonia requiring intensive care unit and mechanical ventilation occurs in critically ill patients [5].

Together with type III IFNs, IFNs-I represent the first line of immune defense against viral infections. In the case of RNA viruses, after recognition of viral products by pattern recognition receptors (PRRs), such as the main cytosolic receptors RNA helicases retinoic acid-inducible gene I (RIG-I) and melanoma differentiation-associated gene 5 (MDA5), the signal converges on the activation of the mitochondrial antiviral signaling protein (MAVS), that, in turns, activates the TANK-binding kinase 1 (TBK1), leading to the phosphorylation and activation of IFN-regulatory factors 3 and 7 (IRF3, IRF7) [6,7]. IRFs then translocate to the nucleus and induce the production of IFNs-I (IFNα, IFNβ, IFNε, IFNτ, IFNκ, IFNω, IFNδ and IFNζ).

Production and secretion of IFN into the surrounding tissue results in the binding of IFN to their receptor (IFNAR) in an autocrine and paracrine manner. The interaction with IFNAR activates the receptor-associated protein tyrosine kinases Janus kinase 1 (JAK1) and tyrosine kinase 2 (TYK2) phosphorylate signal transducer and activator of transcription 1 and 2 (STAT1 and STAT2) molecules, leading to their dimerization, nuclear translocation and binding to IRF9 to form the ISG factor 3 (ISGF3) complex. These events culminate with the transcription of hundreds of interferon stimulated genes (ISGs), that inhibit virus multiplication at distinct levels, potentiate the innate antiviral response and stimulate an adaptive response [7].

Many, if not all viruses, including the human coronaviruses SARS-CoV and MERS-CoV [8,9], have evolved distinct mechanisms to escape immune surveillance, including strategies to avoid PRR recognition and the expression of viral proteins that impair IFN signaling at different levels [9,10]. Therefore, with the experience gained during the previous *Betacoronavirus* outbreaks [8], the IFN response in SARS-CoV-2 infection was promptly investigated. In this review, we focus on viral immune evasion mechanisms observed in vitro and modulation of type I IFNs expression in COVID-19 patients, describing how genetic and autoimmune defects influence disease progression. Finally, an overview of ongoing studies investigating the therapeutic potential of type I IFNs in COVID-19 treatment is provided.

## 2. In Vitro Inhibition of the IFN-I System by SARS-CoV-2

SARS-CoV-2 is an enveloped, positive-sense single-stranded RNA virus, member of the *Coronaviridae* family and *Orthocoronavirinae* subfamily, which includes four genera, α-, β-, γ- and δ- coronaviruses (CoVs). Among the seven CoVs known to infect humans (hCoVs), three of them are the epidemic *Betacoronaviruses* SARS-CoV, MERS-CoV and SARS-CoV-2, while the 229E, OC43, NL63 and HKU1 are endemic hCoVs [11,12]. A phylogenetic analysis of SARS-CoV-2 isolates showed a high similarity (≈96.2%) with the bat coronavirus RaTG13, indicating that it may have originated from bats, such as SARS-CoV and MERS-CoV [1,13,14]; in addition, the high sequence identity with pangolin coronaviruses, particularly in the receptor binding domain (RBD) coding region, suggests that the SARS-CoV-2 ability to bind the human ACE-2 receptor is the result of natural selection [13,15]. The ≈30 kb genome of SARS-CoV-2 shares ≈80% and ≈50% sequence identity with SARS-CoV and MERS-CoV, respectively [11,12,15]. Despite the similarities with SARS-CoV, a higher rate of transmission has been documented for SARS-CoV-2 [16], in part related to the strong binding of the viral spike protein to the ACE-2 receptor [16]. Furthermore, the highest viral load during SARS-CoV-2 infection is observed a few days after the onset of symptoms, whereas SARS-CoV peaked not before the second week of illness. These kinetic differences could explain the fact that SARS-CoV infection was detected before the maximum level of transmission is reached [16].

**Figure 1:**
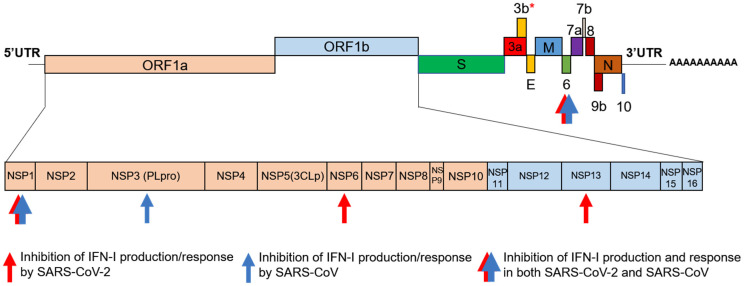
Schematic representation of SARS-CoV-2 genomic organization and gene products. Red arrows, pointed at genes, represent the inhibition of IFN-I production and response determined by the corresponding SARS-CoV-2 gene product. Blue arrows, pointed at genes, represent the inhibition of IFN-I production and response determined by the corresponding SARS-CoV gene product. Combined red and blue arrows, pointed at ORF-6 and NSP1, represent the inhibition of the IFN-I production and the response to IFN-I, determined by the corresponding SARS-CoV and SARS-CoV-2 gene products. * = increased anti-IFN activity in its naturally occurring elongation variant.

The antagonistic activity of viral proteins against the immune system is also crucial for virus replication and spread; SARS-CoV-2 expresses 16 nonstructural proteins (Nsp1–16), 4 structural proteins S (spike), E (envelop), M (membrane) and N (nucleocapsid) and 8 accessory proteins, encoded by ORF3a, ORF3b, ORF6, ORF7a, ORF7b, ORF8, ORF9b and ORF10. While Nsps1–16 are involved in RNA-dependent genomic RNA replication, accessory proteins promote virus infectivity and mediate pathogenic responses (Figure 1).

**Figure 2:**
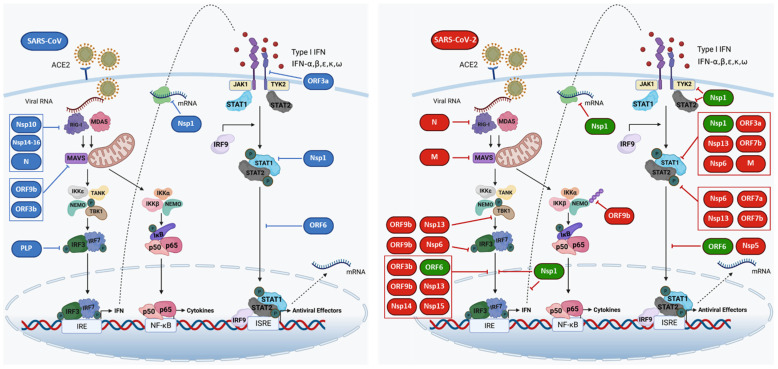
Schematic overview of type I IFN production and response and the counter measures due to the activity of several SARS-CoV-2 (red) and SARS-CoV (blue) proteins. These factors act at different steps in the signal transduction pathway, leading to inhibition of IFN-I production in the first stage and, subsequently, by inactivating the JAK–STAT–ISGF3 pathway, impairing ISGs transcription. ORF6 and Nsp1 represent the main inhibitors of IFN production and signaling (emphasized in green).

Several, if not all, SARS-CoV-2 proteins demonstrate, at minimum, a mild inhibitory activity on IFN-I production and/or IFN-I responses (Figure 2, Table 1). To define which viral products have an impact on the IFN-I system, classical approaches were employed, based on the transfection of different cell lines with plasmids expressing reporter genes driven by IFN-β or ISG promoters, together with expression vectors for non-structural, structural and accessory viral proteins and also infected with RNA viruses or expressing cellular proteins, such as RIG-I, MAVS, TBK-1/IKK-ε and constitutively active IRF-3 5D [17], able to induce IFN-β transcription at different key points in the signaling pathway, or stimulated with IFN-β [18,19,20,21,22]. 

SARS-CoV-2 proteins Nsp6, Nsp13 and ORF7b are implicated in IFN-I evasion by blocking STAT1 and STAT2 phosphorylation, while Nsp1, ORF3a and M inhibit selective phosphorylation of STAT1 [18,22]. Nsp6 and 13 also functioned as inhibitors of TBK-1-mediated IRF-3 phosphorylation and TBK-1 phosphorylation, respectively, with SARS-CoV-2 Nsp6 showing a significant higher ability to block IFN-I production and signaling than MERS and SARS-CoV Nsp6 [18].

ORF3b strongly antagonized IFN-I promoter activation by impairing IRF3 nuclear translocation, with increased activity in its naturally occurring variant, compared with the corresponding SARS-CoV gene product [23]. Together with ORF6, the viral proteins Nsp13, 14 and 15 serve as inhibitors of IFN-β production, demonstrating their ability to interfere with IRF3 nuclear localization [22]; interestingly, when comparing SARS-CoV and SARS-CoV-2 papain-like protease (PLpro) activity, Yuan et al. found that, despite 83% homology in amino acid sequence, SARS-CoV PLpro inhibited IFN-I production and signaling to a greater extent than SARS-CoV-2 PLpro [22].

Also, Nsp12, Nsp14, ORF3 and M proteins produced more than 50% inhibition of IFN-I induction after RIG-I overexpression [20]. However, subsequent studies generated contradictory results about the inhibitory activity of Nsp12 against IFN production and signaling [24,25], indicating that luciferase-based assays may be misleading [24]. The M protein was shown to impair IFN-I promoter activation by interfering with the prion-like aggregation of MAVS and its association with SNX8, thus disrupting the recruitment of the downstream components TRAF3, TBK-1 and IRF3 to the MAVS complex [26]. Moreover, SARS-CoV-2 nucleocapsid protein was shown to repress RIG-I-mediated IFN-β production, possibly through the RIG-I DExD/H domain that possesses ATPase activity [21]. Wu et al. [27], reported that the N-terminal region of ORF9b associates with the antiviral modulator NEMO and interrupts K63-linked polyubiquitination, thereby inhibiting NF-κB signaling and suppressing IFN production and pro-inflammatory cytokines expression [27]. ORF9b also blocks IFN production and signaling by preventing TBK-1 activation and IRF3 phosphorylation and nuclear translocation, thus resulting in the enhancement of viral replication [28]. In a functional analysis of SARS-CoV-2 proteins, Hayn et al. identified different viral components based on their ability to interfere with the three major branches of innate immune response, IFN induction, pro-inflammatory cytokine signaling and autophagy [29]. Repression of type I IFN induction was observed with Nsp1, Nsp3, Nsp5, Nsp10, Nsp13, ORF6 and ORF7b, that interfered to a lesser extent with type II and III IFNs responses. SARS-CoV-2 Nsp5 impaired type I IFN signaling by inducing phospho-STAT1 and phospho-STAT2 accumulation and Nsp14 prevented STAT1 activation by inducing lysosomal degradation of IFNAR1, whereas ORF6 and ORF7b blocked the trafficking of transcription factors and suppressed STAT1 phosphorylation, respectively [18,20,22]. In addition, Hayn et al. described different mechanisms by which viral proteins blocked autophagy flux [29]; overexpression of M resulted in accumulation of LC3B in the perinuclear space, whereas E, ORF3a and ORF7a blocked autophagic turnover. In particular, ORF3a and ORF7a exerted the most potent autophagy antagonism, targeting the late endosome pathway by blocking the fusion of lysosomes with autophagosomes and by decreasing lysosomes acidification, respectively. SARS-CoV-2 Nsp15 was shown to inhibit de novo autophagy induction and, compared to SARS-CoV, was less efficient in blocking type I IFN induction and signaling [29].

Despite such discrepancies—with SARS-CoV-2 viral products producing different results in similar experimental settings—all studies confirmed the role of ORF6 as a potent inhibitor of both IFN-I expression and IFN-I stimulation of ISGs [18,19,20,22]. Comparative experiments with SARS-CoV-2 and SARS-CoV also identified ORF6 as the most consistent inhibitor of the IFN-I system among the two related coronaviruses [18]. ORF6 localizes to the nuclear pore complex (NPC) and exerts its functions by binding to Nup98-Rae1 [30]; this association impairs importin karyopherin alpha (KPNA)2-mediated nuclear translocation of activated IRF3 and ISGF3/STAT1 (Figure 2) [18,30]. A single methionine-to-arginine substitution at residue 58 impairs ORF6 binding to the Nup98-Rae1 complex, thus abolishing its IFN-I antagonism [30].

Nsp1 was identified as another potent viral inhibitor of both IFN-I production and response (Figure 2) [18,20]. Nsp1 inhibited cellular protein synthesis and appeared to act after viral genomic mRNA translation to reduce ribosome pools that engage cellular mRNAs. Cellular RNA translational inhibition thus promoted viral protein synthesis because of the higher efficiency of viral versus cellular 5′ UTRs [31]. In addition, Burke et al. demonstrated that Nsp1 blocks nuclear translocation of IRF3 and found that newly synthetized mRNAs are retained nearby transcription sites during SARS-CoV-2 infection. In addition, the nuclear export of IFN mRNAs is impaired by Nsp1 and, at least in part, by the host endoribonuclease RNase L, which is activated upon infection [32]. The strong antiviral activity exerted by Nsp1 is also supported by another study evidencing that IRF3 phosphorylation and nuclear translocation are compromised in cells overexpressing Nsp1 and that this protein reduces the expression of STAT2 and Tyk2, thereby suppressing type I IFN signaling [33]. Thus, ORF6 and Nsp1 represent preferential targets of therapeutic interventions aimed at relieving the IFN-I blockade during the early stages of infection.

The emergence of SARS-CoV-2 variants could compromise the efficacy of targeted therapies and vaccines. By combining genome sequencing and phylogenetic analysis of SARS-CoV-2 clinical isolates, Lin et al. identified 35 recurrent variants and found the presence of a deletion in the Nsp1 (Δ500–532) in more than 20% of analyzed samples [34]. This Nsp1 variant correlated with lower IFN-β serum levels in patients and an impaired IFN response following in vitro infection of Calu-3 cells [34]. The ability of SARS-CoV-2 variants to evade IFN-mediated immune response is highlighted by the different inhibitory effect elicited by 17 human interferons tested in vitro against 5 viral lineages, belonging from early (lineage A and B) and late (lineages B.1, B.1.1.7 and B.1.351) stages of the pandemic [35]. When comparing IFN-I sensitivity of clinical isolates, it has been observed that, while no differences were detected between lineages A and B, emerging variants B.1, B.1.1.7 and B.1.351 showed from 17 to 122-fold higher resistance to IFN-I than the lineage B and this effect increased up to 25–322-fold, when compared to lineage A [35]. In addition, taking the lineage B isolate as reference, the authors found that the IFNs 50% inhibitory concentrations (IC_50_) was from 4.3 to 8.3-fold higher for IFNβ and from 3.0 to 3.5 higher for IFNλ1 in the lineage B.1.1.7 and from 2.6 and 5.5-fold higher IC_50_ for IFNλ1 and IFNβ, respectively, in the lineage B.1; in addition, in the lineage B.1.351, the IC_50_ further increased up to >500-fold for IFNβ and 26-fold for IFNλ1 [35]. These observations emphasize the importance of type-I IFNs in countering SARS-CoV-2 infection and indicate that the development of mechanisms aimed at eluding the IFN system represent a critical factor in virus evolution.

## 3. Sex Differences, Inborn Errors and Anti-IFNs Auto-Antibodies in COVID-19

SARS-CoV-2 infection has shown a wide range of clinical manifestations, ranging from asymptomatic to life-threatening disease. Different epidemiological factors account for an increased risk of COVID-19 severity, such as being elderly, being male, obesity and the presence of previous comorbidities, including cardiovascular and respiratory diseases, diabetes and cancer [36,37]. These observations indicate that host factors are predictive of disease progression and, together with viral factors, drive the pathogenesis, with interferons (IFNs) playing a crucial role [38,39]. Differences in immunological responses between males and females are well documented [40]. A high percentage (≈80%) of autoimmune diseases occurs in women, while men have higher risk of death from malignancies and show lower antibody response to vaccines. These differences are observed also in infectious diseases. For example, in HIV infection women show lower viral loads but higher risk of progression to AIDS; on the contrary, men show a reduced capacity to rapidly clear HCV during acute infection and are, in general, more susceptible to bacterial infections [40,41]. Collectively, these findings indicate a more robust immune response and a greater ability to control infections elicited by women rather than men.

Several studies report that, in COVID-19, a different clinical outcome occurs in females and males, with a more severe disease, increased odds of ICU admission and higher mortality observed in the latter [36,42,43,44]. These observations could be explained considering a different expression of pattern recognition receptors (PPRs) involved in viral sensing, such as TLR7, which is encoded by the X chromosome and may escape X inactivation in women; also, in response to TLR7 ligands, pDCs production of IFN-α was significantly higher in female cells than in males [40]. Furthermore, severe COVID-19 and higher mortality are associated with a disproportionate inflammatory response and “cytokine storm” in SARS-CoV-2-infected males compared to females, possibly contributing to a worse disease progression and leading to a poor outcome [45].

Takahashi et al. [46] examined cytokine production and immune phenotype in males and females from a cohort of 98 SARS-CoV-2 positive patients. A first analysis was performed on 39 patients, not admitted to ICU, by comparing their immune parameters at baseline with those of healthcare workers (HCWs). A second examination, including a larger cohort of patients, was conducted to assess the differences during the disease course. While no differences between the sexes were observed in the levels of type-I, -II or -III IFNs at baseline, higher levels of IFN-α2 were found in female patients than in male patients in cohort longitudinal analysis [46]. In age- and BMI-adjusted analysis, a higher expression of pro-inflammatory cytokines and chemokines was detected at baseline (IL-8 and IL-18) and in longitudinal analysis (CCL5) in male patients than in females. Notably, a significant correlation was found between CCL5 levels and expression of non-classical monocytes at baseline in male patients; nevertheless, when comparing in a sex-disaggregated manner patients with worsening disease to those with stabilized clinical conditions, a higher expression of CCL5 correlated with a worse disease course in female patients but not in males. Interestingly, a robust T cell response detected in female patients at baseline was not observed in male patients. In addition, disease progression in males was associated with a lower proportion of activated T cell (CD38+ HLA-DR+) and terminally differentiated T cells (PD-1+ TIM-3+). The authors suggest that the differences observed at baseline between sexes underlie different mechanisms of early response to SARS-CoV-2 infection and may regulate disease progression; therefore, these factors should be considered for prognostic and therapeutic sex-dependent approaches. In another study [47], analysis of convalescent plasma from 126 donors with mild or moderate disease evidenced a stronger antibody response and a higher percentage of neutralizing antibodies in male plasma compared to female plasma. The hypothesis is that the more severe disease and the enhanced inflammatory response observed in males could explain a greater B-cell recruitment and antibody production [47]. Conversely, Lieberman et al. [48] found, in male patients, a down-regulation of B cell-specific and NK cell-activating markers and an up-regulation of several inhibitors of NF-κB signaling, in a shotgun RNA sequencing profiling of nasopharyngeal (NP) swabs from 430 SARS-CoV-2 positive individuals. According to the authors, these observations could indicate an inadequate activation of antiviral immunity, or a negative-feedback mechanism triggered in order to reduce excessive inflammation. However, time of sampling should be carefully considered when comparing immune responses observed in different studies.

### 3.1. Inborn Errors in COVID-19

In spring 2020, the COVID Human Genetic Effort [49] and the COVID-19 Host Genetics Initiative [50] were established to elucidate the role of host genetic factors in SARS-CoV-2 susceptibility and COVID-19 severity. Zhang et al. [51] tested the hypothesis that monogenic inborn errors in three loci identified as mutated in patients with life-threatening influenza (TLR3, IRF7 and IRF9) and 10 loci mutated in patients with other viral illnesses, but directly connected to the main three, could also underlie life-threatening COVID-19 pneumonia. Genetic screening of 659 patients with severe COVID-19 relative to 534 individuals with asymptomatic or benign infection revealed an enrichment in functional defective variants at the 13 loci in the first group of patients. In 23 patients (3.5%), autosomal recessive (AR) deficiencies (IRF7 and IFNAR1) and autosomal dominant (AD) deficiencies (TLR3, UNC93B1, TICAM1, TBK1, IRF3, IRF7, IFNAR1 and IFNAR2) were identified and 10 of these patients had low serum IFN-α levels. In vitro experiments showed that cells obtained from patients with AR IRF7 deficiency had impaired production of type I IFN, whereas, in presence of AR IFNAR1 deficiency, they did not respond to IFN-α2 or IFN-β stimulation. Interestingly, none of these 23 patients had never been previously hospitalized for severe viral illness, suggesting that these genetic defects may have a higher penetrance for COVID-19 than other infections. Another study aimed to elucidate genetic alterations underlying interindividual clinical variability [52]; an association was found between the homozygosity for the C allele (CC vs. CT/TT), due to the single-nucleotide polymorphism rs12252, in the interferon-induced transmembrane protein 3 (IFITM3) gene and COVID-19 severity [52]. From the GenOMICC (Genetics Of Mortality In Critical Care) genome-wide association study (GWAS) [53] performed in 2244 COVID-19 critically ill patients from 208 UK intensive care units (ICUs), a significant correlation between disease severity and the presence of variants in or near genes related to antiviral response (OAS1, OAS2, OAS3 and IFNAR2) and inflammatory response (TYK2 and DPP9) was observed [53]. Mendelian randomization and transcriptome-wide association (TWAS) assays revealed a causal link between low expression of IFNAR2, high expression of TYK2 or the monocyte/macrophage chemotactic receptor CCR2 and life-threatening COVID-19 [53]. A whole-exome sequencing identified loss-of-function variants in X-chromosomal TLR7 in four young males admitted to the ICU [54]. These nonsense and missense TLR7 variants impaired type I and II interferon response, as observed with decreased mRNA expression of IRF7, IFNB1 and ISG15 upon pharmacological stimulation of TLR7 in PBMCs isolated from patients [54]. According to these findings, Fallerini et al. detected the presence of TLR7 missense variants in ≈2% of male patients with severe COVID-19, also demonstrating that these variants negatively impact on TLR7 downstream signaling and IFN-related gene expression [55]. Conversely, two studies reported no evidence of an association between rare variants in interferon signaling genes and risk of severe COVID-19 [56,57].

### 3.2. Auto-Antibodies against Type-I IFN in COVID-19

While searching for inborn errors of type I IFN immunity in patients with life-threatening COVID-19 pneumonia [51], Bastard et al. [58] tested the hypothesis that neutralizing auto-Abs against type I IFNs may underlie severe COVID-19. The authors found that 135 of 987 patients (13.7%) with life-threatening COVID-19 pneumonia had IgG auto-Abs against IFN-ω, IFN-α, or against both. These auto-Abs had the ability to neutralize IFNs both in vivo and in vitro in 101 patients (10.2%) and were absent in 663 individuals with asymptomatic or mild infection, whereas present in only 4 of 1227 (0.33%) healthy individuals. Furthermore, of 22 patients with auto-Abs against IFN-α2 tested, all of them also had auto-Abs against all 13 IFN-α subtypes and 2 of them produced neutralizing auto-Abs against IFN-β. The presence of these auto-Abs was associated with a poor outcome, with death occurring in 37 of the 101 patients (36.6%). Substantial evidence indicates that these neutralizing auto-Abs against type I IFNs preceded and were not a consequence of SARS-CoV-2 infection, such as the already known presence of auto-Abs in some individuals and the pronounced excess of male patients (94%) suggesting an X-linked defect, therefore preexisting to infection [58]. In a study from Yale School of Medicine [59], 177 SARS-CoV-2 infected patients and 22 infected HCWs were screened for auto-Abs against extracellular and secreted proteins and results were compared to those of 30 healthy individuals. The Rapid Extracellular Antigen Profiling (REAP) high-throughput platform revealed a higher presence of auto-Abs against immunomodulatory factors, including type I IFNs, in COVID-19 patients than in healthy controls and the number of these auto-Abs positively correlated with disease severity [59]. Finally, longitudinal analysis revealed that, although these auto-Abs were already present in some patients before SARS-CoV-2 infection, a broad subset of auto-Abs (including IL-6, IL-13 and IL-34) was induced following infection [59].

## 4. IFN Expression in SARS-CoV-2-Infected Patients

An early report by Blanco-Melo et al. [60], conducted using serum samples from SARS-CoV-2 positive patients upon admission to the hospital and from post-mortem lung biopsies, demonstrated a marked dysregulation of genes involved in innate and humoral immune responses, expression of pro-inflammatory cytokine (e.g., IL-6) and chemokines, whereas no variation in IFN-α/β and type III IFNs levels was detected [60]. Reduced IFN production [39,61] and a decrease in their transcript levels, together with a marked reduction of ISG15 and ISG56 expression in oropharyngeal swab samples [62], were observed in critically ill patients requiring invasive mechanical ventilation. These findings support the notion that coronaviruses may profoundly interfere with IFN response without inhibiting the inflammatory NF-κB cascade [63,64]. In vitro experiments on SARS-CoV-2-infected cells identified several viral proteins as potential inhibitors of IFN signaling, such as ORF3b [23], ORF6, ORF8 and nucleocapsid protein [19].

Although no changes in IFNs levels were detected in a transcriptomic sequencing analysis on cells obtained from bronchoalveolar lavage fluid, a prominent induction of ISGs (e.g., IFIT/IFITM genes, ISG15, RSAD2 and IRF7) and chemokines (e.g., CXCL1, -2, -8 and CCL2, -7) were observed in these samples and their levels were closely associated with viral load [65]. A comprehensive immunophenotyping study reported a type I IFN response coupled with TNF/IL-1β-driven inflammation in classical monocyte from severe COVID-19 patients; the hyperinflammatory signature associated with PMBCs differed from that observed in patients with severe influenza [66]. In a recent study, Zheng et al. reported that the viral E protein can be sensed by TLR2 and promote inflammation [67]; mouse bone marrow-derived macrophages (BMDMs) and human PBMCs stimulated ex vivo with purified E protein, increased the production of several pro-inflammatory molecules, including IL-6, TNF-α and Il-1β. Furthermore, in vivo administration of E protein induced inflammatory cells recruitment and lung tissue damage in wild type mice but not in Tlr2^−/−^ mice. In addition, treatment of SARS-CoV-2-infected mice with TLR2 inhibitors reduced the release of pro-inflammatory cytokines and increased survival, suggesting a potential use of TLR2 inhibitors in therapy [67]. Overall, the increase in monocyte and neutrophil counts, together with marked lymphopenia, have been widely reported during COVID-19 [65,68,69,70,71]. The prominent release of monocyte, neutrophil and other immune cells chemoattractants promotes pro-inflammatory cells migration into the lungs; consequently, the inflammatory environment established in the lower respiratory tract tissue may favor viral cell entry [72] and worsening tissue damage that, in turn, may contribute to the Acute Respiratory Distress Syndrome (ARDS) pathogenesis [73]. Over-representation of immune cells, including eosinophils, seems to be associated with more severe illness. Lucas et al. [74] identified a “core COVID-19 signature” of inflammatory cytokines, which persisted at high levels only in severe patients. In contrast to the observations mentioned above showing blunted IFN responses, severe cases shown a sustained cell-associated and plasma IFN-α/IFN-λ expression at the later stages of disease, while IFN expression decreased after 10 days from symptom onset in moderate patients [74].

Interestingly, a differential expression among isoforms of type I IFN has also been reported. While IFN-β levels remained stable, a higher expression of IFN-α was observed in lung and upper airways compartments [75] and in individuals who needed intensive care unit treatment [76]. Regardless of disease severity, significant upregulation of type I IFN and IFN-λ1, -λ2 and -λ3 expression [62] but not type II IFNs [77] were found in cells of the upper respiratory tract, suggesting a differential induction of the IFN transcriptome. Furthermore, SARS-CoV-2 shares, with the other coronaviruses, the ability to interrupt antiviral protein translation into infected cells as an immune escape strategy [31]. While the precise timing of action is still debated, human ex vivo evidence showed an imbalance between IFN-λ1 and IFN-λ2 mRNA and protein levels in nasopharyngeal samples; transcripts amounts were strongly associated with the viral load but not accompanied by an increase in protein levels [78].

The activity of type II IFN (IFN-γ) must also be considered in the context of SARS-CoV-2 infection; type II IFN overlaps with the functions of type I IFNs in countering virus propagation and is also recognized as a crucial immunomodulatory cytokine for the development of the adaptive immune response to infection, thereby reducing immunopathology [79]. Reduced expression of IFN-γ by circulating CD4^+^ T cells has been associated with higher levels of pro-inflammatory cytokines, such as IL-6 and TNF-α, in severe compared to moderate cases [80]. On the other hand, elevated serum IFN-γ production was detected in individuals after admission to the intensive care unit (ICU), compared to healthy control subjects. The specific enhancement of TGF-β expression in severe cases suggested the potential use of this cytokine as a predictive factor of disease severity [81]. However, IFN-γ upregulation was observed within 3–10 days from symptoms onset in the cells of the upper respiratory tract from symptomatic cases [82], suggesting its involvement in the antiviral response since the early stages of the disease.

## 5. IFN-Based Therapy for COVID-19

Given the urgent need for an effective treatment for patients suffering from COVID-19, the number of registered clinical trials increased significantly in the past year; most studies were primarily designed to evaluate the efficacy and safety of treatment with compounds already approved for clinical use. The dysregulation of type I IFN response commonly observed during coronaviruses infection [8,60,83] and the high sensitivity of SARS-CoV-2 to IFN-I identified through in vitro experiments [84,85] raised interest in strategies based on these cytokines. Further indications in favor of the clinical use are provided by the encouraging results obtained from the treatment of diseases etiologically linked (or not) to viral infection (reviewed in [86]).

Several clinical trials have been conducted and are still ongoing to examine the potential use of different IFN-I subtypes and routes of administration for improving the clinical outcome of patients infected with the new coronavirus (Table 2). 

An early open-label phase 2 study of mild-to-moderate COVID-19 patients showed that treatment with subcutaneous injection of IFN-β1b, within 7 days from symptoms onset, combined with oral lopinavir-ritonavir and ribavirin was safe and superior to therapy without IFN administration in shortening the time to symptoms alleviation, the duration of SARS-CoV-2-RNA positivity tested in nasopharyngeal swab and hospital stay period. Besides, no significant adverse events were reported [87]. In addition, the combination IFN-β1b plus lopinavir/ritonavir and ribavirin was investigated in a single center observational study showing lower 28-day mortality (9% vs. 12%) and less need for systemic corticosteroids, as compared to favipiravir (FPV)-treated individuals in a cohort of hospitalized patients with non-critical COVID-19 [88].

Previously reported as a candidate agent for the treatment of SARS-CoV infection [89], IFN-β1a administration was also investigated in a similar setting including lopinavir-ritonavir or atazanavir-ritonavir plus hydroxychloroquine in COVID-19 cases. In this randomized clinical trial of 81 patients, the subcutaneous administration of IFN-β1a in severely ill patients resulted in significantly lower 28-day mortality and increased discharge rate on day 14. Interestingly, analysis based on the time of treatment initiation showed greater efficacy in mortality reduction when IFN was administered early during the disease evolution [90]. The importance of administration timing has been highlighted by a recent report in which delayed IFN-β administration in MERS-CoV-infected mice exacerbated a pro-inflammatory state and increased infiltration of activated monocytes, macrophages and neutrophils in the lung, ultimately resulting in a worse outcome (e.g., fatal pneumonia), compared to mice treated within one day after infection [91]. Thus, the IFNs response timing relative to the virus replication seems to be a critical factor that may profoundly affect the disease course. Although obtained on a small number of patients (*n* = 20), further data support the use of IFN-β1a, hydroxychloroquine and lopinavir/ritonavir for the management of COVID-19 [92]. Conversely, in the DisCoVeRy phase III trial (NCT 04315948) the lopinavir/ritonavir plus IFN-β1a arm (145 adults hospitalized for COVID-19) did not show clinical improvement at day 15 nor viral clearance in respiratory tract specimens, while hospital discharge at day 29 was significantly higher than the control arm (HR, 0.72; 95% CI, 0.54–0.96; *p* = 0.026) [93].

In order to reach an adequate concentration in the upper and lower respiratory tracts and limit systemic exposure to IFN, other routes of administration were also evaluated. Nasal drops of recombinant human IFN-α provided a valuable prophylactic measure in individuals at high risk of infection. An experimental trial of 2944 healthcare workers in Hubei (China), compared to new-onset COVID-19 in healthcare workers in the same Province (including Wuhan), showed that the 28-day incidence of COVID-19 and the incidence of new-onset clinical symptoms with negative images for pneumonia, were zero in the treated group [94]. Furthermore, treatment with nebulized IFN-α2b, with or without Umifenovir (Arbidol), was tested on of 77 confirmed COVID-19 patients. In this exploratory study, Zhou et al. [95] reported a significant reduction in the duration of detectable SARS-CoV-2 RNA in the upper respiratory tract concurrently with reduced duration of high IL-6 and C-reactive protein circulating levels [95]. Another promising approach via nebulization involves the use of IFN-β1a (SNG001). Results from a phase II trial, marked by a strong odds reduction (79%) of developing severe disease or dying in SNG001-treated patients than in the placebo groups [96], have recently led to recruitment for a randomized, double-blind, placebo-controlled, phase III trial to determine the efficacy and safety for the treatment of hospitalized patients who require oxygen supplementation (ClinicalTrials.gov: NCT04732949).

## 6. Conclusions

During the past year, it has become clear that an enormous heterogeneity exists in the magnitude and kinetics of the early innate immune response during SARS-CoV-2 infection, suggesting that a dysregulated and/or delayed IFN response are likely associated with a poor prognosis. An accurate disease status definition, the consideration of inherent genetic defects and comorbidities that could affect the IFN response against viral infection may provide new insights and foster a better understanding of IFN response during SARS-CoV-2 infection.

Recent genetic observations also highlight the association between severe COVID-19 outcomes, rare genetic variants and/or presence of auto-Abs, both impairing type I IFNs signaling. This scenario could have important clinical implications; detection of genetic defects or auto- Abs in SARS-CoV-2 infected patients could be used as a prognostic factor of severe disease; also, these patients could undergo personalized therapy to decrease the concentration of anti- type I IFNs auto-Abs, as already demonstrated in four patients [97]; finally, an IFN-based therapy could be considered. Nevertheless, while beneficial in the early phase of infection when the antiviral activity of IFNs limited SARS-CoV-2 replication, a detrimental response may be elicited in late stages, when uncontrolled IFN response could drive inflammatory lung pathology.

The encouraging findings obtained to date from ongoing clinical trials indicate that administration of type I IFN may represent a valuable strategy to combat COVID-19 at early stages of disease. Further investigations are necessary to develop targeted therapies according to the disease severity, bearing in mind the importance of the innate response as a first-line immune defense against viral infection.

## Figures and Tables

**Table 1:** Antagonism of Type-I IFN response by SARS-CoV-2 proteins.

SARS-CoV-2 Protein	Cellular Target	Mechanism	References
Nsp1	40s ribosomal subunit; STAT1; IRF3; STAT2; Tyk2	Translation inhibition by interfering with host mRNA binding; blocks IRF3 nuclear translocation; blocks nuclear export of host mRNAs; inhibition of STAT1 phosphorylation; reduced expression of STAT2 and Tyk2	[18,31,32,33,34]
Nsp5	STAT1; STAT2	Induces phospho-STAT1/2 accumulation imparing type I IFN signaling	[29]
Nsp6	IRF3; STAT1; STAT2	Inhibition of IRF3, STAT1 and STAT2 phosphorylation	[18]
Nsp13	TBK1; IRF3; STAT1; STAT2	Inhibition of TBK-1, STAT1 and STAT2 phosphorylation; blocks IRF3 nuclear translocation	[18,22]
Nsp14	IRF3	Inhibits IRF3 nuclear translocation; induces lysosomal degradation of IFNAR1	[22,29]
Nsp15	IRF3	Inhibits IRF3 nuclear translocation	[22]
ORF3a	STAT1	Inhibits STAT1 phosphorilation	[18]
ORF3b	IRF3	Inhibits IRF3 nuclear translocation	[23]
M	MAVS; STAT1	Impairs MAVS aggregation and recruitment of downstream components; inhibits STAT1 phosphorilation	[18,26]
ORF6	IRF3; ISGF3	Blocks IRF3 and ISGF3 nuclear translocation	[18,19,20,22,30]
ORF7a	STAT2	Inhibits STAT2 phosphorilation	[18]
ORF7b	STAT1; STAT2	Inhibits STAT1 and STAT2 phosphorilation	[18]
ORF9b	NEMO; TBK-1; IRF-3	Interrupts K63-linked polyubiquitination and inhibits NF-κB signaling; blocks activation of TBK-1 and IRF-3	[27,28]
N	RIG-I	Binds to the DExD/H domain and represses IFN-β production	[21]

**Table 2:** IFN-based treatment studies.

Authors	IFN Therapy	IFN Administration	Type of Study	N. Patients	Disease Stage	Outcome (Intervention vs. Control)
Hung, I.F.-N. et al. [87]	IFN-β-1b 5 days from symptoms onset	Subcutaneous	Multicentre prospective open-label randomized phase 2 Trial	86 intervention group 41 control group	Hospitalized	Hospitalization: 9 vs. 14.5 days Mortality: 0% vs. 0% Serious adverse effects: 0% vs. 2%
Malhani, A.A. et al. [88]	IFN-β-1b 4 days from symptoms onset	Subcutaneous	Observational study IFN-based vs. FPV treatment	68 treated with IFN 154 treated with FPV	Mild–moderate–severe	Mortality: 9% vs. 12% Need of systemic corticosteroids: 57% vs. 77%
Davoudi-Monfared, E. et al. [90]	IFN-β-1a 10 days from symptoms onset	Subcutaneous	Open-label randomized clinical trial	42 intervention group 39 control group	Severe	Hospitalization: 14.8 vs. 12.2 days Mortality: 19% vs. 43.6% Serious adverse effects: no differences between groups
Dastan, F. et al. [92]	IFN-β-1a 6.5 days from symptoms onset	Subcutaneous	Prospective non-controlled trial	20 intervention group only	Severe	Hospitalization: 16.8 days Mortality: 0% Serious adverse effects: 0%
Ader, F. et al. [93]	IFN-β-1a 10 days from symptoms onset	Subcutaneous	Open-label randomized adaptive clinical trial	145 intervention group 148 control group	Moderate–severe	Hospital discharge at day 29 significantly higher than control arm
Meng, Z. et al. [94]	Recombinant human (rh) IFN-α Preventive Therapeutic Strategy	Intranasal	Prospective, open-label study	2944 intervention group only	None	28-day incidence of COVID-19/new-onset clinical symptoms: 0% Serious adverse effects: 0%
Zhou, Q. et al. [95]	IFN-α2b 8 days from symptoms onset	Inhaled	Uncontrolled, exploratory study	53 intervention group 24 control group	Moderate	Accelerated viral clearance/reduction in systemic inflammation markers (circulating IL-6 and CRP levels)
Monk, P.D. et al. [96]	IFN-β-1a 24 h from SARS-CoV-2 positive test	Inhaled	Randomized, double-blind, placebo-controlled, phase 2 pilot trial	50 intervention group 51 control group	Moderate–severe	Greater odds of improvement in OSCI scale for intervention group Mortality: 0% vs. 6% Serious adverse effects: 15% vs. 28%

IFN, Interferon; rhIFN-α, Recombinant human IFN-α; IL-6, Interleukin 6; CRP, C-reactive protein; OSCI, Ordinal Scale for Clinical Improvement.

## Data Availability

Not applicable.
